# Early Marriage and Barriers to Contraception among Syrian Refugee Women in Lebanon: A Qualitative Study

**DOI:** 10.3390/ijerph14080836

**Published:** 2017-07-25

**Authors:** Zeinab Cherri, Julita Gil Cuesta, Jose M. Rodriguez-Llanes, Debarati Guha-Sapir

**Affiliations:** 1 Centre for Research on the Epidemiology of Disasters, Institute of Health and Society, Université Catholique de Louvain, 1200 Brussels, Belgium; giljulita@gmail.com (J.G.C.); debarati.guha@uclouvain.be (D.G.-S.); 2 Directorate D—Sustainable Resources, Joint Research Centre, European Commission, Ispra (VA), 21027 Varese, Italy; jose-manuel.rodriguez-llanes@ec.europa.eu

**Keywords:** Syria, Lebanon, refugees, refugee health, public health, war, conflict, reproductive health, barriers to healthcare, marriage, adolescents, adolescent marriage

## Abstract

The Syrian conflict has displaced five million individuals outside their country with Lebanon hosting the largest numbers per capita. Around 24% of Syrian refugees fleeing to Lebanon are women of reproductive age (15–49). Yet, a better understanding of the sexual and reproductive health needs of Syrian refugee women in Lebanon is required to improve provided services. Eleven focus group discussions were conducted in four regions of Lebanon with 108 Syrian refugee women of reproductive age. Thematic analysis was used to examine the data. Interviewed women were mainly adults. They believed that, in Lebanon, they were subjected to early marriage compared to the norm in Syria due to their financial situation and uncertainty. Cost was reported as the main barrier to use contraception in Lebanon but some Syrian refugee women were not aware of free services covering sexual and reproductive health. In general, marriage, pregnancy, and family planning behavior of Syrian refugee women in Lebanon slightly differed from those in Syria pre-conflict in terms of age of marriage, conception subsequent to marriage, and contraception method. Hence, interventions to increase awareness of subsidized sexual and reproductive health services, including free contraceptives at primary health care centers, and those targeting protection from early marriage of Syrian refugee women in Lebanon are strongly recommended.

## 1. Introduction

Worldwide forced displacement increased in 2015 with a record of 65.3 million displaced individuals by conflict and persecution [1]. The Syrian conflict increased the global number of refugees whereby five million Syrians have fled the country as of March 2017 [2]. According to the United Nations High Commissioner for Refugees (UNHCR), Lebanon hosts the largest number of Syrian refugees per capita, more than one million individuals, accounting for more than a fifth of the country population before the crisis [3]. Unlike other massive population displacements characterized by refugees mainly living in camp settings, the Government of Lebanon refused the formal establishment of camps. Consequently, by 2016, Syrian refugees are dispersed all over Lebanon, living in informal tented settlements (ITS) or renting homes and apartments [4,5]. The highest numbers have been found in the governorate of Bekaa (360,733) followed by Beirut and Mount Lebanon (BML) (287,651) [6].

Worldwide, forced displacement and conflict lead to death of a yearly 140,000 women [7] and to loss of livelihoods, increased disease transmission risk and poverty, and disturbance of vital services, including sexual and reproductive health (SRH), family planning, and antenatal care [8,9]. Even after displacement, SRH needs of women may further worsen due to lack of routine services. Around 24% of Syrian refugees fleeing to Lebanon are women aged 18 to 59 [3]. Women living in conflict areas and as refugees are more prone to poor SRH [10,11,12,13,14,15,16], which increases the likelihood of their mortality and morbidity [17,18,19]. Conflict-affected or refugee women tend to have decreased accessibility to contraceptives and limited financial resources [20,21]. Although having access to family planning enables refugees, specifically women, to take crucial decisions related to their SRH, during conflict, many of them have limited control over decisions related to family planning [20,22]. By allowing women to postpone their first pregnancy and have safe intervals between pregnancies, family planning could prevent nearly a third of the roughly 300,000 yearly maternal deaths around the globe [22,23]. If there is a three-year interval between successive children, an additional 1.6 million children younger than five would survive [22]. Moreover, poverty in the family, insecurity, and risk of sexual violence contribute to child marriage which, in turn, is linked to complications during pregnancy, insufficient antenatal care, preterm labor, and low birth weight [24,25,26].

Sexual and reproductive health (SRH) rights and needs, including comprehensive family planning services, of refugee women are recognized by various international charters, such as the program of action of the International Conference on Population and Development [27] and the Minimum Initial Service Package established by the Sphere Project and applied by numerous international organizations [28]. In Lebanon, international and local Non-Governmental Organizations (NGOs) provide SRH care for Syrian refugee women (SRW) primarily through primary health care centers (PHCC) by offering free or heavily subsidized family planning services (insertion of intrauterine device, contraceptive pills, and condoms); consultations with a midwife or Obstetrician/Gynecologist; and laboratory and diagnostic tests [29]. Nevertheless, recent studies done in Lebanon indicated that SRW are not aware of these subsidized services (37.8%), identified different barriers to SRH services including contraception, and found that 42.3% of SRW had not been utilizing any type of contraception before pregnancy [23,30]. The studies’ results suggest that forced migration and displacement led to decreased contraception use due to difficulty in finding and using effective methods of contraception. On the other hand, few if no published literature examining early marriage of SRW in Lebanon exists. Only one study reports the mean age of first marriage as 19.0 ± 4.0 [30].

In spite of the growing evidence of the impacts of displacement and conflict on SRH, family planning services provision in conflict settings remains inadequate [29], and the priorities for care and needs among refugees are yet quite unknown [19]. The studies about SRH of Syrian refugees are still limited in Lebanon and the region [22,30] and do not examine in depth the SRH behavior of SRW in Lebanon in terms of contraceptive use and barriers, early marriage practices and reasons, and perception towards appropriate age of marriage. Knowing that the Syrian refugee population is dynamic, their needs are changing quickly, and the need to understand their perceptions, the objective of this study was to assess the SRH needs and behavior of SRW, with a focus on marriage perceptions and behavior, in order to improve the services provided and their acceptability.

## 2. Materials and Methods

### 2.1. Study Design

Focus group discussions, a qualitative method, allow comparisons of different opinions on sensitive subjects which single individuals cannot fully grasp, including SRH problems [31]. Although qualitative studies provide more in-depth understanding, are quick to organize and are not costly, they provide enlightening evidence in conflict-related humanitarian settings [31]. We conducted a total of 11 focus groups in different governorates of Lebanon (see Table 1) where Syrian refugees reside including BML, South, North and Bekaa during May 2016. Six ITS and five PHCC were included. The study was done in collaboration with the International Medical Corps (IMC), an international organization which seeks to improve the lives of vulnerable communities through the provision of health care and related services. Based on the literature review on SRH and the information required by the assessment of IMC, we utilized a semi-structured focus group discussion guide (see Appendix A), adapted from a previous guide administered by IMC in 2015 in the PHCC and ITS where they provide services. Each focus group addressed the following topics: marriage practices of Syrians, pregnancy behavior and perceptions of SRW, family planning behavior and perceptions including barriers. We translated the guide to Arabic and piloted it twice; once in the North and once in the South of Lebanon. At the end of the focus group, we collected demographic information (age, marital status, number of children, educational level, religion, year of arrival to Lebanon, and Governorate of origin in Syria) of the SRW.

The performance and distribution of focus groups was decided based on the request of the IMC health team in Lebanon as part of its assessment and regular monitoring activities of services provided to SRH of SRW in Lebanon.

### 2.2. Study Population

We targeted 100 SRW of reproductive age (15–49) living in Lebanon since 2011 or later according to their oral statement. We excluded women not belonging to the previously mentioned age group or who had been living in Lebanon before March 2011. Due to IMC’s operational needs, the dynamic and complex context of Syrian refugees in Lebanon and taking into account the difficulties to accessing SRW, we used a convenience sample. The latter included women attending PHCC or living in ITS who receive services from IMC. The Community Health Workers working in the PHCC and ITS, to provide SRW with information about health services, recruited women according to the previously mentioned inclusion and exclusion criteria. Focus groups included women of mixed ages, number of children, and marital status. 

We recruited a total of 165 women. A total of 108 women participated in the eleven focus groups. Initially, 114 women agreed to participate, 6 women withdrew their participation during the discussion because they felt uncomfortable or because they were called by their husbands.

### 2.3. Study Setting

Data collection was approved by IMC after revision of study protocol and guide, as part of its assessment and monitoring activities of services provided to sexual and reproductive health of SRW in Lebanon. A researcher, native Arabic speaker (Zeinab Cherri), conducted the focus groups in a safe and private place secured by IMC. The researcher thoroughly read the consent form (see Appendix B) to the SRW explaining the purpose behind the study and emphasizing their right to withdraw at any time. We maintained confidentiality at all times and did not collect names or any identifying information. SRW had the freedom to leave the focus group at any point. All SRW gave verbal consent to participate before the focus group discussions were conducted. We used verbal informed consent rather than a written one because we believed it would create a less formal situation facilitating a more open discussion. Based on the ethical criteria of the American Anthropological Association, the consent “does not necessarily imply or require a particular written or signed form. It is the quality of the consent, not its format, which is relevant” [32]. SRW did not receive any monetary incentive and their partaking in the study was voluntary. IMC served refreshments in the ITS and distributed female kits (soap, pads, towel) in the PHCC as in other regular activities.

### 2.4. Data Analysis

Zeinab Cherri transcribed verbatim the focus group discussions in colloquial Arabic to preserve the meaning of the interviewee’s statements. Consequently, we did not use a qualitative software for analysis since the one accessed in our institution, NVivo, cannot analyze data in Arabic. Instead, we inductively analyzed the focus groups in Arabic language using concrete steps starting with open coding for each subsequent transcript. Since Zeinab Cherri is the only researcher who could write and read Arabic, she performed the thematic analysis by combining the coded themes which emerged from open-coding. The list of themes was then discussed to reach consensus among the authors. Throughout the process, we continuously reviewed the themes until no further theme was identified. Possible relations between each of the themes were explored. Quotes extracted and used in this manuscript were translated to English [33].

## 3. Results

We present our results according to the life-cycle of SRW, beginning with their demographic characteristic and followed by their marriage, pregnancy and family planning behavior.

### 3.1. Characteristics of SRW

The demographic characteristics of the interviewed SRW in four governorates in Lebanon are presented in Table 2. Overall, the age distribution of SRW was as follows: 22.2% were between the ages of 19 and 24, and 48.2% were between the ages of 25 and 35. The proportion of women aged 36 to 49 was 19.4% and only 10.2% were between 15 and 18 years. The majority of the women were married (91.7%), and 63.6% of SRW aged 15 to 18 were married. Their number of children was as follows: 35.2% had one to two children, 21.3% had three to four children, 15.7% had five to six children, 6.5% had more than six children, and 21.3% did not have children at all. The majority of the women had elementary education in school (40.7%). Only 4.6% reached a university level and 7.4% did not receive formal education at all. Refugee women had their original homes in various regions of Syria. They came from 9 of the 14 Syrian governorates, mainly Homs (21.3%) and Daraa (19.4%). Arrivals of SRW started as soon as 2011, and peaked during 2012 (53.0%), the early phases of the conflict; yet many women in our sample arrived in 2013 (17.6%) and 2014 (15.7%). A smaller share reported coming to Lebanon in most recent years.

### 3.2. Marriage Practices and Perceptions

**Figure 1:**
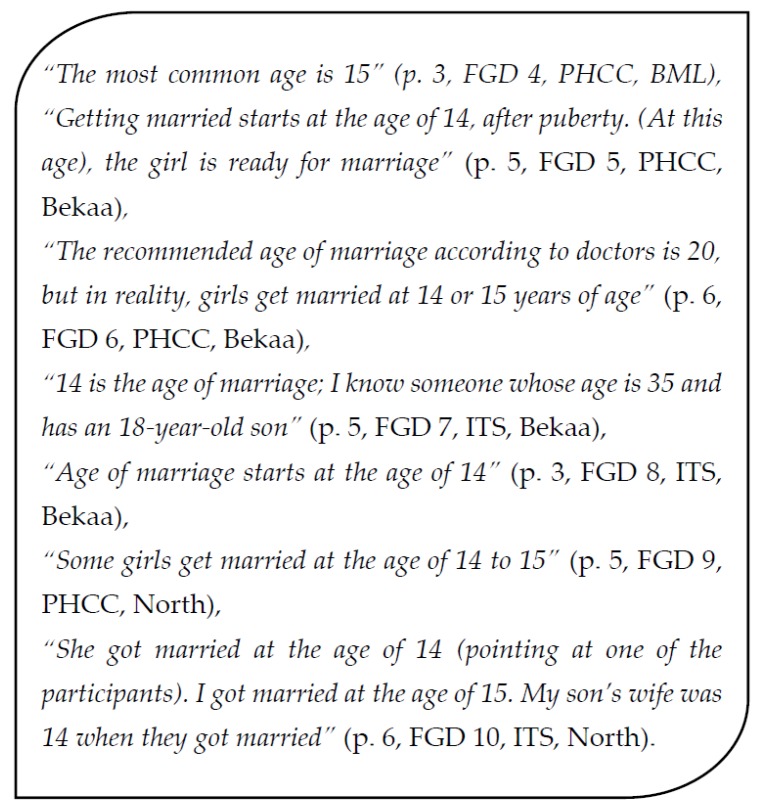
Quotes about age of marriage from selected focus group discussions (FGD); PHCC: primary health care center; ITS: informal tented settlements.

When SRW got married in Syria, the majority of interviewed women claimed that the most common age of marriage was 14 to 15 years old, and that most girls would get married before the age of 20, after which the girl would have been considered a spinster. Women from different focus groups expressed this opinion (Figure 1).

Education was a reason to delay marriage for girls to the age of 18–19 until they finish high school. They could continue their studies at university, but while being married. However, if girls, were not pursuing their secondary education, they should directly get married. SRW believed that this behavior has not changed in Lebanon, and others thought that it might be happening at a slightly higher rate due to the absence of schooling opportunities for Syrians in Lebanon. A woman who got married at the age of 19 in Lebanon narrated her story, “*I (got married) at the age of 19, but I had not finished my schooling. We left Syria in 2012. I kept waiting to return to Syria to continue my Baccalaureate till 2014. Then what? Returning to Syria was not possible; and I could not continue my education here, (so) yes I got married*” (p. 11, Focus Group Discussion (FGD) 11, ITS, BML).

Some women believed that the fact of marrying at a young age (14–15) has increased in Lebanon due to their poor living conditions and conflict in Syria. Two major drivers were noted, insecurity and poor financial situation in Syria. Parents were even sending their daughters from Syria to get married in Lebanon to help them escape the conflict. One woman who was sent from Syria to Lebanon to get married referred to herself as a real-life example saying: “*I am a real life example (as) I did not know my husband. I hadn’t seen him until we got married*” (p. 5, FGD 7, ITS, Bekaa). The women further explained that parents had fears in Lebanon and believed that marriage would protect their daughters. Parents feared their daughters might be attacked or raped, and as a result, no man will accept to marry them. Another reason parents had was financial. Syrian families in Lebanon consisted of large numbers and parents wanted to relieve a bit of the responsibility off of their shoulders. If the girl got married, it would be the responsibility of the husband to protect her and take care of her financially. 

When asked about the appropriate age for girls to get married, the majority of women believed that it should be 18 and above whether in Syria or Lebanon. They clarified that getting married at a young age, girls have a weak back and are immature causing health problems and leading to marriage problems between the couple. One woman pointed out: *“(Before the age of 18), the girl has a weak back that does not handle pregnancy leading to abortion.”* (p. 6, FGD 6, PHC, Bekaa) while the other elucidated: “*Of course, first of all, (young girls) have a small uterus. If she is pregnant at a young age, she will suffer from problems in the uterus*.” (p. 6, FGD 9, PHCC, North). Another woman stated: “*(a young girl) does not know how to take responsibility or take care of her husband or child. Sometimes, (they) would stay in conflict for two to three years until they understand each other well sometimes (this) does not happen leading to divorce*.” (p. 3, FGD 2, ITS, South). Another woman expressed her opinion on the issue saying that by getting married at a young age, parents were increasingly burdening their daughters who were already suffering from the conflict. Her sister, for example, got married at the age of 14 for one month and then got divorced because she was not able to adapt to life with a man. 

### 3.3. Pregnancy Behavior and Perceptions

Syrian couples maintained similar pregnancy behaviors and perceptions as the ones they had in Syria but some differences were identified. Newly married Syrian couples did not intend to wait before having their first child and did not use any contraceptive method at the beginning of marriage. Otherwise, the husband’s family would believe there was something wrong with his wife and would motivate him to marry a second wife in order to have children. In multiple focus group discussions, women expressed their belief that using a contraceptive method before being pregnant for the first time was harmful to the woman who might, as a result, become infertile; moreover, delaying pregnancy is not her decision to take. In addition, women cannot convince their husbands to wait for a certain period of time before getting pregnant, as the husbands prefer to have children directly. One woman explained: “*(Getting pregnant) directly is better. (If her pregnancy) got delayed by two months (after marriage), she is a bit late. A woman cannot take any contraceptive or family planning method when she first gets married. She does not have the will to delay pregnancy. She (also) cannot agree (with her husband) to wait for four months or one year before getting pregnant; no (she cannot).*” (p. 6, FGD 5, PHCC, Bekaa)*.* In exceptional cases, women were able, in agreement with their husband, to wait for a year before getting pregnant. After having three to four children, women could discuss the use of contraception with their husbands.

Women believed that the appropriate duration of time to wait, in Syria, before getting pregnant was one to two years for the couple to enjoy their time together alone and for them to know each other well, thus decreasing the chances of divorce. A woman said: “*In my opinion, (the woman) should wait for a year, so that the wife and husband understand each other, and for her to recognize whether this man deserves to bear her children or not, and if she can live with him the rest of her life or not. Hence, a one-year probation is better.*” (p. 6, FGD 6, PHCC, Bekaa)*.* Women thought that young girls (14–15) should wait until they become 18 before getting pregnant to protect their health and to become more capable of raising a child, considering that at their age, they were children themselves. In Lebanon, particularly, SRW believed that it is a necessity for newly married couples to wait before having children to consider their financial situation and the process of registering the child. Some women believed that there should not be any waiting time and the best approach was to directly get pregnant. They also believed that it was their right, regardless of their current situation, to have children and they were annoyed from the pressure they are subjected to in Lebanon by the society or healthcare professionals who comment on their fertility; *“In Lebanon, they always relate Syrians to reproduction and pregnancy and they say Syrians reproduce a lot whether in the community or in PHCC”* (p. 6, FGD 1, PHCC, South)*; “I only had one child and wanted to have another one. The gynecologist kept asking me about the reasons for having another child since I already have one. She told me that you, Syrians, are only good at reproducing”* (p. 6, FGD 11, ITS, BML). 

SRW stated that Syrian couples were used to having a lot of children. The number of children stated differed with the generation interviewed. Older generations of women aged forty and above reported having eight to twelve children. Younger generations of women aged less than forty had four to five children. Women believed that the appropriate number of children was four to five, but taking into account their current situation in Lebanon, this number decreased to one or two, with some husbands even accepting this number. An interviewee explained: “*The situation in Syria is different from here. In Syria, it is normal to give birth and (let the children grow) freely. The country is ours and the situation is known there, but here no (it is different). If any (bad thing) happens to the parents here, what happens to the children? Hence, (having) less children is better.*” (p. 4, FGD 3, ITS, South). Some exceptional women thought that the appropriate number was still six children in Lebanon and expressed their love of having a lot of children and raising them. Syrians were thinking of the many difficulties faced in Lebanon before having children including their financial status, children’s registration, education, needs and upbringing, pregnancy and delivery costs, and the uncertainty to which they are subjected. Some women cited their own health and their personal life as reasons not to have many children.

### 3.4. Family Planning Behavior and Perceptions 

Our studied Syrian women in Lebanon were using contraceptive pills and injectable contraception (every three months), intrauterine device (IUDs), withdrawal (pull out method), rhythm method, and breastfeeding as birth control methods. The most used methods were pills and IUDs; whereas, women believed fertility, the rhythm and withdrawal methods were the best and most harmless approach of birth control. The majority of women said that they were using their preferred contraceptive method. Some women stated that their preferred method, injectable contraception, was not available in Lebanon. Other women complained about the quality of IUDs in Lebanon (made of steel) and believed that ones in Syria (made of silver) were better. A different group of women were not able to use IUDs because of their price (USD 66.67 (Conversion from Lebanese Pound to United States Dollar is as follows: 1500 LBP = USD 1) to USD 100) in private clinics. These women were not aware that IUDs were given for free in PHCC and only had to pay USD 2 for placement, claiming that IUDs were placed for free in Syria under the patronage of “The Family Planning Unit”.

Barriers to using contraceptive methods differed among SRW in Lebanon. These barriers were mainly similar to the ones faced in Syria, except for cost. Those who were not aware of their availability for free at PHCC believed that, as refugees in Lebanon, cost is a barrier. Several women believed that the husband’s refusal prevented them from using a contraceptive method whether in Lebanon or Syria, while families’ interference played a role to a lesser extent in Lebanon than Syria because married couples were living in/migrated to Lebanon alone. For married women who did not have children and wanted to use birth controls, they would not be able to do so without agreeing about it with their husbands. When asked about religion, all women believed that it was not a barrier. A small number of women considered the side effects and incompatibility of birth control methods to their bodies as barriers not to use them. For instance, women reported that IUDs caused excess bleeding, menstrual irregularities, back pain, and depression; pills led to depression, anger, headache, loss of temper, anxiety, and hypertension; and injectable contraception contributed to hormonal irregularities, bleeding, menstrual irregularities, and anger. Consequently, a very small number of women chose to discuss the use of condoms with their husbands, having the final decision, to relieve themselves of such side effects for a period of time. One participant stated: “*If the woman does not want to use a (contraceptive method) that messes with her hormones, (she suggests the condom to her husband). The final decision is taken by the husband. He either uses a condom or he does not.*” (p. 10, FGD 10, ITS, North). However, some women did not even know what a condom was, and those who knew, stated that it was not common at all because it was recent and not accepted by their husbands who were the main barriers to using it. Some women knew that condoms were available for free at PHCC while other women stated that it was available at pharmacies, but did not know its price.

PHCC were the place to get contraceptive pills and IUDs for women who were aware about their availability there. Other women went to pharmacies to buy contraceptive pills or to private doctors to insert an IUD. At PHCC, pills and IUDs were provided for free, but the cost of IUD insertion was USD 2. At pharmacies, the price of pills varied from USD 3.3 to USD 14 every month. IUD insertion at midwives cost USD 20 to USD 33.3 and at private clinics USD 66.6 to USD 100. Women did not know about the cost of injectable contraception in Lebanon. In Syria, women who use the services of the “Family Planning Unit” claimed that all these services were for free.

In order for women to use contraceptive methods, they first had to agree about using it mainly with their husbands. If their husbands did not accept the idea, it was out of the question. In some cases, families played a role in this decision. If their husbands agreed, they had to decide and search for the best method to use. Women relied on doctors as the main trusted source for information concerning birth control methods. They consulted doctors to know more about which method was most suitable for their cases. Other sources of information in Lebanon included community health workers, pharmacies, family, friends and neighbors. Women based their decision on the best method depending on its price, durability, and side effects. When seeking the birth control method, women could go alone, but they preferred being accompanied by someone. Reasons included possibility of fainting after IUD insertion and feelings of embarrassment when asking for the method alone. Consequently, women took their husbands, family members and neighbors with them to PHCC, pharmacies or private clinics. 

In case women got pregnant, but did not want their pregnancy, participants believed that there was nothing to be done because religion forbade abortion and it was not an available option in Lebanon. One woman said: “*No, it is forbidden (to abort). When a woman is pregnant, that’s it. There is nothing she could do.*” (p. 6, FGD 8, ITS, Bekaa). Some women expressed their desire to have abortion, but could not do it in Lebanon because no doctor accepted to perform the operation. A very small number of women had heard of the emergency contraceptive pill and were able to state its use in case of rape; whereas, the vast majority did not recognize it, though it was available free-of-charge at PHCC.

## 4. Discussion

Our study suggests that early marriage might have increased due to the situation of Syrian refugees in Lebanon, most importantly unveiling several of the reasons underlying this pattern and SRW’s perceptions on these issues. Women believed that the age of 18 is the minimum appropriate age of marriage for girls, and were aware that early marriage and pregnancy can have negative consequences, increasing early divorce and on reproductive health, respectively. Despite their understanding of these consequences, the climate of uncertainty and financial constraints as refugees were perceived as the roots of the upward trend in early marriage. On the other hand, in Lebanon, similar to the situation in Syria pre-conflict, SRW had the same beliefs concerning conception, preferring to directly get pregnant after marriage. However, the harsh conditions in Lebanon are making some of them rethink this decision and even the appropriate number of children. SRW women were using different types of contraceptive methods in Lebanon; already having children is a main condition for the couple to agree on using contraception, and participants expressed cost as the main barrier for use. Some SRW remain unaware of the free-of-charge SRH services provided to them in the different PHCC in Lebanon and few others were not aware of the condom and its uses.

In the literature, an increase in early marriage has been reported during periods of conflict among affected individuals [34]. A United Nations International Children’s Emergency Fund (UNICEF) study assessed the rate of child marriage among marriages registered with Jordan’s Sharia courts. Despite the fact that such a publication does not provide a perfect estimate of the frequency of this issue [35], it provides indication of its change over time in the population of Syrians in Jordan. In two years of conflict, the rate of early marriage doubled from 12% (2011) to 25% in 2013. The perceptions amongst the interviewed SRW in our study were convergent with the quantitative data from Jordan. Moreover, in Save the Children’s report “Too Young To Wed”, Syrian parents reported financial sponsorship of daughters as a main reason for letting their under-18 daughters get married in Jordan [36]. This is similar to what we found in this study in which SRW reported that Syrian refugee parents are more likely to wed their young girls in Lebanon due to financial reasons and harsh living conditions, especially with the absence of formal camps. Other reasons mentioned by SRW were parents’ fear due to insecurity and lack of educational opportunities. In Lebanon, 69% of Syrian refugees reported feeling insecure due to verbal or physical harassment [37], and two thirds of Syrian refugee children are not receiving education [38]. Islam was not brought up by any interviewee as a driver of early marriage, which is neither encouraged nor condemned by any religion [39].

SRW identified the age of 18 as an appropriate age of marriage, clarifying the health and social dangers to which girls, marrying at a young age, could be subjected. This is a very important finding showing that SRW are aware of the consequences of early child marriage. Based on the findings discussed above, interventions targeting early marriage of SRW should focus on empowering girls with education and their families with financial stability; and both of them with a sense of security during their stay in Lebanon.

The majority of Syrian women believed that the appropriate number of children for couples living in Lebanon should be less than the usual number they had in Syria. Factors playing a role in this decision and pertaining to the situation in Lebanon included education, financial status, children’s registration, needs and upbringing, pregnancy and delivery costs, and the uncertainty to which they are subjected.

Syrian couples who were newly married preferred to directly have children. One reason behind that was the belief of some women that the use of contraceptive methods prior to the first pregnancy could lead to female infertility. This is a common misconception, as a comprehensive review of 17 studies in the literature showed that pregnancy rates after discontinuation of contraception (pills, injections, IUDs, or implants) were similar to rates after discontinuing barrier methods or not using contraception at all [40]. Hence, awareness given to SRW should address this misconception.

Whether in Lebanon or Syria, married Syrian women with no children cannot use contraceptives without their husband’s approval. This is similar to Jordanian couples whereby the final decision on issues related to family planning is left, in the majority of the cases, to the husband as the male head of the household [41].

Studies show that Middle Eastern displaced women face a lot of obstacles when accessing family planning services, especially conservative norms and patriarchy [13,42]. On the other hand, a needs assessment, conducted by United Nations Population Fund (UNFPA) in 2012 in the North and the Bekaa with Syrian displaced women, found that almost 74% wanted to prevent pregnancy and identified high cost, unavailability of desired type of contraceptive [43], and fear as the main barriers to contraceptive use [30,43]. SRW women in our study who were not aware of free family planning services, identified cost as the main barrier; especially because they were used to free family planning services provided in Syria [23,44]. Other obstacles were similar to the ones faced in Syria and religion was not considered a barrier for married women, knowing that Muslim scholars consider the use of contraception acceptable during marriage as long as the couple agrees on it [45]. Nevertheless, the norms of patriarchy allow husbands to have the final decision on the use of contraception, permits parents’ interference, and forces women to succumb to such decisions and interferences [46]. The findings about early marriage and contraception from this study provide evidence refuting the myth that Muslim communities are strictly conservative. When it comes to unmarried women, however, the situation is different as sexual relationships outside wedlock are considered a solid religious taboo among Muslim communities [39].

Some women were also unaware of condoms and its use, while women who were aware of it stated that their husbands resist using it, a phenomenon also exhibited with Jordanian men [41]. This calls for special attention to males’ resistance when planning to deliver family planning services. Moreover, similar to our study, in the UNFPA assessment, IUD, oral contraceptives and the rhythm method were used by women, a result consistent with pre-conflict Syrian 2006 national statistics [30].

A 2006 study conducted by the Syrian Ministry of Health suggested that the majority of women who performed abortion in Syria, though illegal, sought medical doctors using safe methods [44]. In the UNFPA study, 52.1% of SRW in Lebanon did not want their current pregnancy. Similarly, a few women in our study wanted to abort pregnancies in Lebanon, but faced obstacles: the belief that it is forbidden in religion and inability to find abortion services. This falls out of the scope of international organizations addressing the Syrian crisis in Lebanon because it is still considered illegal [46]. Nevertheless, the desire of women to abort and reasons behind it should be considered as an area of future research and advocacy.

The main limitations of our study were that the selected areas where focus groups had been conducted were chosen by IMC according to their convenience to facilitate recruitment and increase participation. Women were recruited by IMC local health staff (IMC local health staff included Lebanese health officers and Syrian community health workers) who may have recruited women who had more access to IMC services. Focus groups included women of different age groups and marital status; this might have introduced potential bias in the answers of single and younger women. Although the discussions were conducted in Arabic and transcribed in Arabic, some bias may still have occurred during results analysis in English. Moreover, relying on self-report may be subject to over- or under-reporting. Although confidentiality was maintained, some of the discussed topics might have been a taboo issue for women who did not fully express their opinions. 

Despite these limitations, we gained rich and diverse qualitative data parallel to the objective of the study intended to increase our understanding of the different SRH issues Syrian refugee women are facing without generalizing the results for all Syrian refugee women in Lebanon. 

## 5. Conclusions 

This study provides a complex and thorough understanding of the sexual and reproductive health needs, preferences, behavior and barriers for Syrian women who are refugees in Lebanon; and indicates that their conflict-related experience partially changes their behavior and perceptions. SRW in Lebanon mainly have similar marriage, pregnancy, and family planning behaviors as the ones they had in Syria. However, as refugees in Lebanon, SRW believe that they are subjected to early marriage at a higher level than in Syria because of their parents’ fears and financial constraints, and that they should have less children due to their situation of uncertainty. SRW believe that the appropriate age of marriage or pregnancy is 18 and above, taking into account the young girl’s health and education. Syrian refugee women mainly use IUDs and contraceptive pills. Despite services covered by international and local NGOs, some SRW remain unaware of the free services provided in PHCC in Lebanon or about condoms. The results of this qualitative study could be used to construct a quantitative survey assessing SRH behavior and needs of SRW in Lebanon. This survey and other research is needed to advocate for more funding targeting SRH services of SRW in Lebanon. In addition, following are recommendations to be considered by the government and international organizations working with SRW in Lebanon:

1. The Lebanese government and UNHCR could collaborate to increase educational opportunities for Syrian refugee girls in Lebanon to protect them from early marriage.
2. UNHCR could allocate part of the funds targeting Syrian refugees in Lebanon to help Syrian refugee families gain economic independence as another protection from early marriage.
3. IMC and other partner organizations could work on:
  - increasing the knowledge of availability of free contraceptive methods including condoms at PHCC for SRW, and addressing misconceptions about contraception;
  - recruiting and training SRW to increase the knowledge of other SRW regarding available SRH services in PHCC;
  - recruiting and training male Syrian refugees to organize awareness sessions about condoms for other Syrian males in ITS or PHCC.

## Figures and Tables

**Table 1:** Distribution of focus group discussions in all governorates of Lebanon by setting.

Setting	BML	South	North	Bekaa	Total
Informal Tented Setting	1	2	1	2	6
PHCC	1	1	1	2	5
Number of Women	19	30	19	38	108

BML: Beirut and Mount Lebanon; PHCC: primary health care centers.

**Table 2:** Characteristics of interviewed Syrian refugee women in four governorates in Lebanon.

Characteristic of SRW	Region in Lebanon				Total (%)
	BML	South	North	Bekaa	
Age					
15 to 18 years	1	1	1	8	11 (10.2%)
19 to 24 years	3	7	7	7	24 (22.2%)
25 to 35 years	11	16	8	17	52 (48.2%)
36 to 49 years	4	6	5	6	21 (19.4%)
Marital Status					
Single	1	1	1	6	9 (8.3%)
Married	18	29	20	32	99 (91.7%)
Number of Children					
0	1	4	4	14	23 (21.3%)
1 to 2	6	15	8	9	38 (35.2%)
3 to 4	6	6	5	6	23 (21.3%)
5 to 6	5	4	2	6	17 (15.7%)
>6	1	1	2	3	7 (6.5%)
Educational Level					
None	0	4	0	4	8 (7.4%)
Elementary	8	11	11	14	44 (40.7%)
Preparatory	5	12	8	12	37 (34.3%)
Secondary	3	3	0	8	14 (13.0%)
University	3	0	2	0	5 (4.6%)
Year of Arrival to Lebanon					
2011	1	3	0	3	7 (6.5%)
2012	12	10	11	24	57 (53.0%)
2013	2	6	5	6	19 (17.6%)
2014	2	6	4	5	17 (15.7%)
2015	1	4	0	0	5 (4.6%)
2016	1	1	1	0	3 (2.8%)
Governorate of Origin in Syria					
Aleppo	1	1	3	1	6 (5.5%)
Ar-Raqqah	1	2	0	12	15 (13.9%)
Damascus	2	1	2	3	8 (7.4%)
Deir ez-Zour	2	4	0	0	6 (5.6%)
Daraa	4	4	10	3	21 (19.4%)
Hama	1	10	0	1	12 (11.1%)
Al-Hasaka	0	0	0	7	7 (6.4%)
Homs	6	7	3	7	23 (21.3%)
Idleb	2	1	3	4	10 (9.3%)

BML: Beirut and Mount Lebanon.

## Appendix A: Focus Group Guide

**Study on the Sexual and Reproductive Health of Syrian Refugee Women in Lebanon Focus Group Discussion with Syrian Refugee Women**

I am interested in learning about some of the needs and concerns of Syrian refugee women related to sexual and reproductive health as well as family planning. I hope that your engagement in the discussion by answering the questions will help improve the available services. I expect our discussion to last about one-and-a-half hours.

*A. To begin with, how long have you been living in this community? In this community, where do women usually seek reproductive health care such as services related to maternal and child health?* *Probe*: *What attracts them to this site specifically? How long is the waiting time to see a doctor?*
*B. Now, I want to ask some questions about marriage.*
  1. What age do people usually marry in your community in Syria?
  2. Has the common age of getting married changed for people who have been displaced from their homes? If yes, why has this change occurred?
  3. How soon after getting married do people have their first child? Probe: what is a good amount of time after marrying to have a child?
  4. Do people think that there is a good number of children to have? What do you think? Probe: why is it good or not to have this number of children? Finances? Family life? Education? Health? Future plans? Anything else?
  5. Thinking about these questions, do you think this is the same here as it was in your home country/setting? Probe: what has changed? Why is it different? Is it different in a good way?
*C. Now, we will discuss birth control methods in this community after you have been settled here.*
  1. What do women in this community do to prevent or postpone having babies? Probe: modern methods, including pills, condoms, and IUDs. Which methods are most popular and why?
    - Where do you find trusted sources of information about family planning?
    - What methods of birth control are available to you in this camp/area?
    - Are there any methods that you would like to use that are not available to you? If yes, what are these methods?
    - Are there any barriers to accessing birth control if you want to use it? If yes, please describe:
    - Are there any costs for these services? If yes, please describe the costs:
    - What can women do if they have unprotected sex and they do not want to get pregnant?
    - What do women do in this community if they are pregnant but do not want to be pregnant?
    - Have you ever heard of emergency contraception? If yes, what have you heard?
    - Do women go alone to get family planning or do they take someone with them? Probe: do women go with husbands? Or mother? Mother-in-law? Anyone else?
    - Is there any contraceptive method that you were using before leaving Syria and not using now? If yes, why?
    - Are there any difficulties in obtaining family planning methods here? What are those difficulties? Probe: staff attitude, opening times, requirements for paperwork —marriage certificate, permission or health records?
  2. How do women in this community make decisions regarding family planning?
    - Why do they choose these methods?
    - How do they make the decision to use these methods? (On their own? With their husband? Following the instructions of their husband? Following the advice of other members of their family? Following the advice of their friends or other community members?)
    - Are there any methods that they would like to use that are not available to them? If yes, what are these methods?
    - Thinking about these questions, do you think this is the same here as it was in your home country/setting? Probe: what has changed? Why is it different? Is it different in a good way?
  3. Are there places in this location where condoms can be found easily? IF YES 9a&b. IF NO 10c.
    - If yes, are the condoms free?
    - If yes, how have women learned about where these condoms can be found?
    - If no, if condoms cannot be found easily, what barriers prevent easy access to the condoms? Probe: what can be done to make condoms more accessible?
  4. Since you have been living here, have any reproductive health supplies been distributed to women or girls?
    - If yes, what supplies specifically? (Probe: menstruation hygiene management supplies, clean delivery kits, family planning) what did the community think about these distributions? Who did the distribution and when?
    - If no, what reasons have you heard for not having these supplies?
*D. Before we finish, I would like to invite you to speak up if there anything about health care services, especially as it relates to reproductive health care changes or care for women and young girls since you arrived to Lebanon, that we have missed and you would like to discuss.*
  1. Do you have any questions?

We thank you all for your time and ideas. This has been extremely helpful. You have all helped to provide a good understanding of the situation here. Your contributions are greatly appreciated. If you have any concerns, or think of additional information that should be shared, you can share with me separately.

Thank you for your help.

## Appendix B: Informed Consent Form

**Consent to Participate in a Study on the Sexual and Reproductive Health of Syrian Refugee Women in Lebanon: Assessment of Barriers to Family Planning**

*Title of the Study:*

Study on the Sexual and Reproductive Health of Syrian Refugee Women in Lebanon: Assessment of Barriers to Family Planning

Sponsor of the Study:

International Medical Corps (address: Sodeco square, Beirut, Lebanon)

*Research Organization:*

Université catholique de Louvain, (address: Promenade de l’Alma, 31 bte B1.41.03 B-1200 Bruxelles)

*Local Investigator:*

Zeinab Cherri, MSc. In Public Health Candidate, Faculty of Public Health, Université Catholique de Louvain, Email: zeinab.cherri@student.uclouvain.be

Hello. My name is Zeinab Cherri. I am a Msc. in Public Health candidate in the Faculty of Public Health, at the Université Catholique de Louvain. I would like to invite you to participate in a research study on sexual and reproductive health behavior of Syrian refugee women (SRW) in Lebanon. The study will examine the knowledge and experiences of SRW in sexual and reproductive health and use of contraceptive methods. Furthermore, the study will describe the general barriers to family planning faced by SRW and those specific to their context now.

Before we begin, I would like to take a few minutes to explain why I am inviting you to participate in this focus group discussion (FGD) and what will be done with the information you provide. You will be asked to answer open-ended questions about pregnancy-related decisions and your knowledge about contraceptive methods. I will also ask you to describe your use of contraception including the barriers that you faced/might face. In most of the questions, I will ask you to think about your current situation and how it might have affected your experience. Finally, I will ask you about your opinion in SRH sessions that you received and whether it had influenced your behavior or not. Please stop me at any time if you have questions about the study.

I am doing this study as part of my thesis research at UCL. I will conduct 10 FGDs with 100 SRW aged 15–49 living in Lebanon since 2011 or later. The information I collect from these discussions will be written in a report and shared with the International Medical Corps but without mentioning any of the participants’ names. I will also use the information in my thesis and academic articles. Your name or any identifying information about you will not be used in any report or publication, and we will protect your privacy and the confidentiality of the information you provide.

The focus group should take approximately one hour to one hour and a half. Please know that your participation is entirely voluntary and your refusal to participate will not result in any loss of benefits. Also, you have the right to withdraw your consent or discontinue the discussion at any time without penalty. There are no foreseeable risks or discomfort but there may be unforeseeable risks such as experiencing distress due to remembering negative experiences. If you feel uncomfortable at any time, you can leave the discussion or you can ask to skip any question that you do not wish to answer. You will be provided with refreshments and a hygiene kit. By participating in this study, you may be helping others to better understand the concerns of SRW women about family planning. In the long term, you may help the IMC understand how to improve its provision of SRH services and awareness sessions. You will not be provided with any payment for your participation.

I would like to record this discussion on a digital recorder to make sure that I accurately capture all the information you provide. I will transcribe the recorded discussions and they will only be used by the research team to analyze the data. The recordings themselves will not be shared with anyone outside the research team. They will be deleted after transcription is complete.

I will give you now some time to make sure that you have read the consent and are sure about wanting to participate. If you have any questions, you are free to ask them now. If you have questions, concerns or complaints about this research study later, you may contact me (Zeinab) at 76-972445; email: zeinab.cherri@student.uclouvain.be

Are you interested in participating in this study?

□ Yes □ No

Do you consent to recording the interview?

□ Yes □ No

Do you allow me to quote from the interview without using your name?

□ Yes □ No

Name of Interviewer: ____________ Signature: ____________ Date & Time: ____________
